# 0101. Early and severe impairment of lactate clearance in endotoxic shock is not related to liver hypoperfusion: preliminary report

**DOI:** 10.1186/2197-425X-2-S1-P12

**Published:** 2014-09-26

**Authors:** P Tapia, D Soto, A Bruhn, T Regueira, N Jarufe, L Alegria, JP Bachler, F Leon, C Vicuña, C Luengo, G Ospina-Tascón, J Bakker, G Hernandez

**Affiliations:** Pontificia Universidad Católica de Chile, Facultad de Medicina, Departamento de Medicina Intensiva, Santiago, Chile; Pontificia Universidad Católica de Chile, Facultad de Medicina, Departamento de Cirugía Digestiva, Santiago, Chile; Universidad de Chile, Hospital Clínico, Unidad de Pacientes Críticos, Santiago, Chile; Fundación Valle del Lili, Intensive Care Unit, Cali, Colombia; Erasmus MC University Medical Centre, Department of Intensive Care Adults, Rotterdam, Netherlands

## Introduction

Although the prognostic value of persistent hyperlactatemia in septic shock is unequivocal, its physiological determinants are controversial. In particular, the role of impaired hepatic clearance has been considered as relevant only in severe shock with liver ischemia or advanced cirrhosis. However, very few studies have addressed this subject.

## Objectives

To determine the evolution of lactate clearance [1] in an endotoxic sheep model.

## Methods

**Figure 1:**
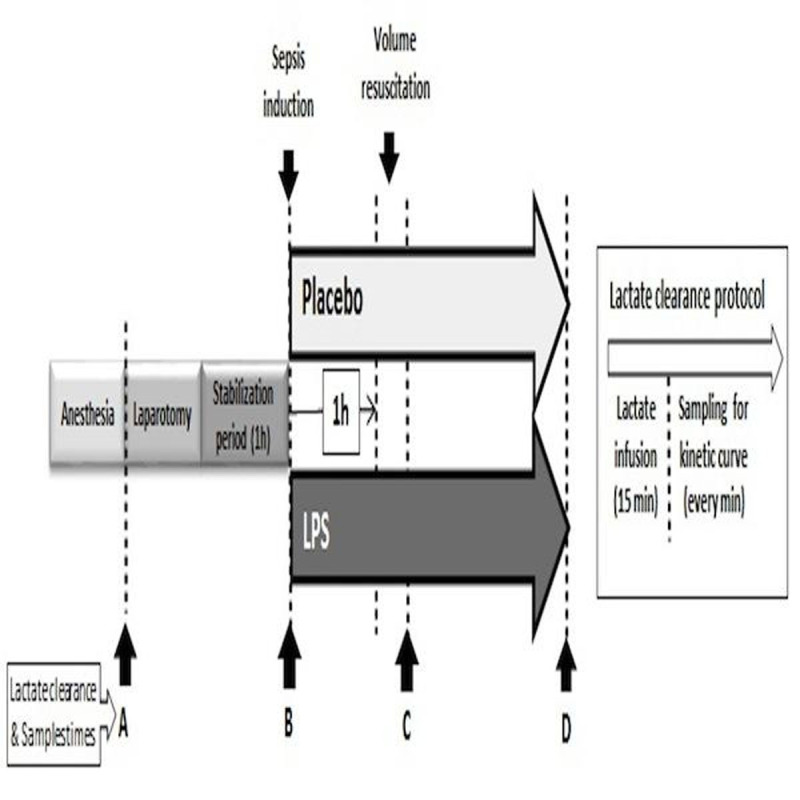
Figure 1

This study is part of a major project exploring the influence of adrenergic stimulation and blockade over the determinants of lactate production and utilization in septic shock. Eight anesthetized sheep subjected to a multimodal hemodynamic/perfusion assessment including pulmonary artery, hepatic and portal vein catheterizations, portal/hepatic artery flow, gut tonometry, sublingual microcirculation, muscle microdialysis and hepatic mitochondrial high-resolution respirometry, were randomized to LPS or sham. LPS sheep received 5 mcg/kg bolus (E coli O127:B8®) and then 4 mcg •kg-1•hr-1 for the rest of the experiment [2]. After 1h they were volume resuscitated. Sampling and exogenous lactate clearances were performed at 4 points (fig 1)

## Results

**Figure 2:**
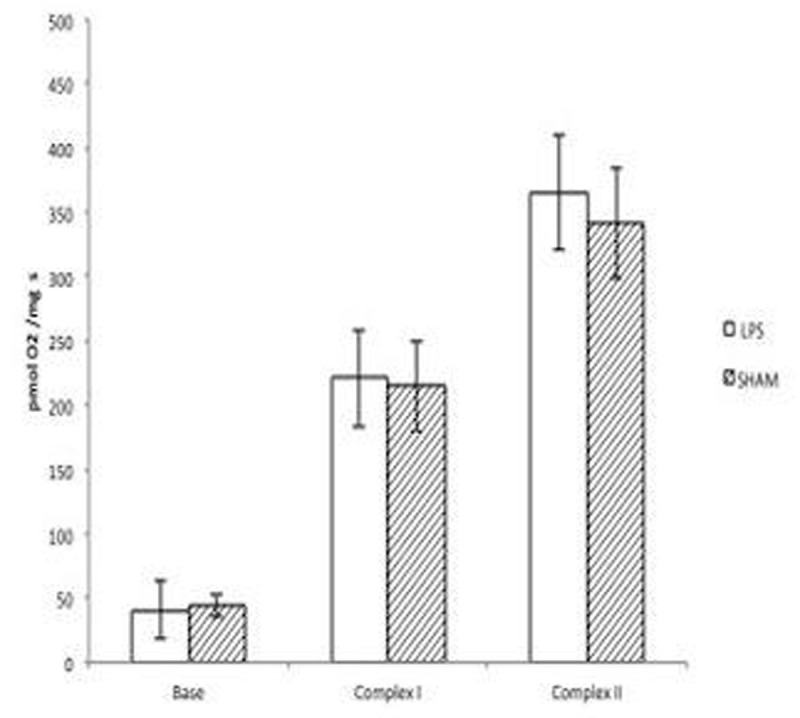
Figure 2

LPS sheep presented an early hyperlactatemic hyperdynamic septic shock and an increased muscle lactate production compared to sham (MAP 63 vs 87 mmHg; MPAP 20 vs 10 mmHg; CO 3.7 vs 2.5 l/min; arterial lactate 6.1 vs 2.6 mmol/l). Total liver flow (865 vs 692 ml/min), proportion of liver flow/CO, O_2_ liver extraction, liver enzymes, and mitochondrial function were comparable between LPS and placebo (fig 2)

**Figure 3:**
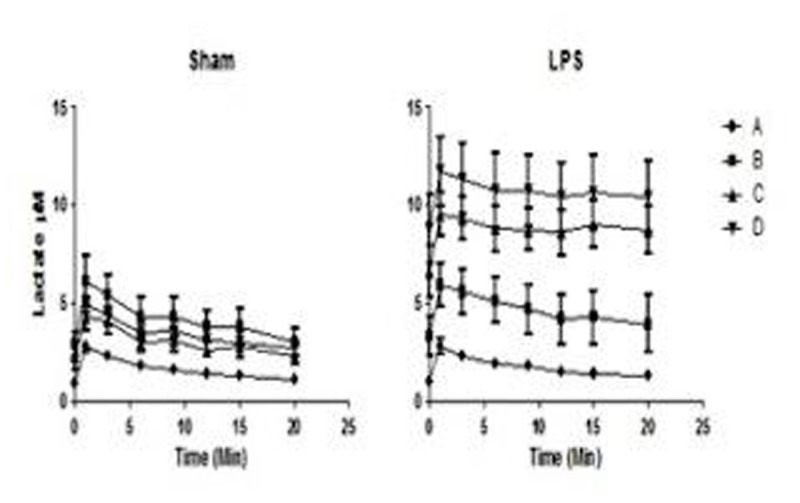
Figure 3

However, LPS sheep presented an early severe and persistent decrease in lactate clearance (C: 575 vs 1188 and D: 907 vs 1410 ml/kg/h), fig 3

## Conclusions

Hyperdynamic endotoxic shock induces an early, severe and persistent impaired lactate clearance that is not related to liver hypoerfusion, O_2_ extraction or mitochondrial function.
